# Facilitation of Health Plan‐Clinic Partnerships to Improve Colorectal Cancer Screening for Rural Medicaid Enrollees: A Qualitative Study

**DOI:** 10.1111/jrh.70203

**Published:** 2026-08-10

**Authors:** Erin S. Kenzie, Anders Herreid‐O'Neill, Emily Myers, Jennifer Coury, Brittany Badicke, Gloria Coronado, Melinda M. Davis

**Affiliations:** ^1^ Oregon Health & Science University – Portland State University School of Public Health Portland Oregon USA; ^2^ Oregon Rural Practice‐Based Research Network Oregon Health & Science University Portland Oregon USA; ^3^ Complex Systems Program Portland State University Portland Oregon USA; ^4^ University of Arizona Cancer Center Tucson Arizona USA; ^5^ Department of Family Medicine Oregon Health & Science University Portland Oregon USA

## Abstract

**Purpose:**

Mailed fecal immunochemical test (FIT) and patient navigation for follow‐up colonoscopy have been shown to increase colorectal cancer (CRC) screening rates, but implementation barriers exist in rural settings. Partnerships between clinics and Medicaid health plans may address these barriers. In a large pragmatic randomized trial, we conducted a qualitative analysis to identify ways in which practice facilitation supported the implementation of a partnered health plan‐clinic intervention of mailed FIT and patient navigation to improve CRC screening among rural Medicaid enrollees.

**Methods:**

In the SMARTER CRC pragmatic trial, study team practice facilitators supported rural clinics and Medicaid health plans in implementing mailed FIT and patient navigation for follow‐up colonoscopy. Qualitative data collected during the first year of implementation included clinic and health plan interviews, clinic contact logs, and monthly periodic reflections with the facilitator team.

**Findings:**

Practice facilitation was needed to support both clinics and health plans in the study. Facilitators supported continued engagement of clinics and health plans, used workflow mapping to align implementation between clinics and health plans, provided technical support for sharing of patient data, and facilitated communication between clinics and health plans. Prior study team experience implementing CRC screening interventions enabled successful facilitation.

**Conclusions:**

Practice facilitation can successfully support partnered health plan‐clinic implementation of mailed FIT and patient navigation in rural clinics. Our findings highlight the importance of assessing support needs across all partnering organizations when implementing multilevel preventive care interventions and underscore the importance of subject matter expertise in practice facilitation.

**Trial Registration:**

ClinicalTrials.gov Identifier: NCT04890054.

## 1 Introduction

Timely and consistent screening is critical for reducing morbidity and mortality from colorectal cancer (CRC) [1], but rates remain low, particularly for people residing in rural communities [2, 3, 4] and for those receiving Medicaid coverage [3, 5]. Common barriers to completing colonoscopy, an effective CRC screening modality, include inability to take time off from work, lack of transportation, stigma, and hesitancy about bowel preparation [6, 7]. Prior research has shown that some of these barriers are more common in rural areas and for Medicaid enrollees with lower incomes [8].

Mailed fecal immunochemical testing (FIT) and patient navigation for follow‐up colonoscopy have been shown to increase screening rates by addressing some of these known barriers [9]. A pilot study conducted by our team, which was a partnership between academic researchers, a community‐based organization, a Medicaid health plan, and primary care clinics, found that mailed FIT was feasible and acceptable for these organizations and supported CRC completion [10]. The study additionally highlighted a need for navigating patients to the follow‐up colonoscopy after abnormal FIT. Combining mailed FIT with patient navigation has also been supported by microsimulation modeling [11] and a systematic review [4].

While mailed FIT and patient navigation can reduce patient‐level barriers to CRC completion, the logistical coordination necessary for these programs is substantial and poses barriers, especially for smaller, rural clinics [12, 13]. For example, population‐based reporting to identify those due for CRC screening can be challenging in primary care clinics with fewer central staff and limited electronic health record (EHR) capabilities [14, 15]. Partnering with health plans has been shown to help overcome these barriers when health plans cover the cost of mailing and support patient identification and vendor coordination; however, this model was previously tested in urban settings and with larger clinic populations [12, 16].

To improve rates of CRC screening in rural Medicaid populations, the SMARTER CRC study used practice facilitation as a meta‐strategy to support a partnered health plan‐clinic intervention involving mailed FIT and patient navigation [16]. Main findings from the SMARTER CRC intervention found that the approach was effective in increasing screening rates, with 7.3% more people screened in intervention clinics compared with control [9]. For this intervention to be adopted in other settings, it is important to understand how implementation strategies, including practice facilitation, can support this partnered health plan‐clinic model. To address this need, we conducted a qualitative analysis of data from interviews, clinic contact logs, and periodic reflections in the first year of the SMARTER CRC study.

## 2 Methods

### 2.1 Study Overview

SMARTER CRC is part of the National Cancer Institute (NCI) Accelerating Colorectal Cancer Screening and Follow‐up through Implementation Science (ACCSIS) Program (NCI Clinical Trials Reporting Program: NCI‐2021‐01032) [17]. The aim of ACCSIS is to conduct multisite, coordinated, transdisciplinary research to evaluate and improve CRC screening processes using implementation science strategies. SMARTER CRC engaged three Oregon Medicaid health plans and 28 rural or frontier clinics in a large‐scale, parallel, cluster‐randomized pragmatic trial [9, 18]. To be included, clinics were located in a geographic region designated as rural or frontier by the Oregon Office of Rural Health or had a Rural‐Urban Commuting Area (RUCA) code of 4 or higher [19, 20, 21]. In the first year of the intervention, clinics were randomized either to implement mailed FIT outreach and patient navigation for follow‐up colonoscopy for abnormal FIT or to continue providing usual care. The intervention was available to all clinics in the second year. In this paper, we focus on the first year of implementation.

The SMARTER CRC intervention was tailored for rural Medicaid populations using Boot Camp Translation and was delivered as a partnership between health plans and clinics, supported by the study team [22, 23]. The study was conducted by the Oregon Health & Science University's Oregon Rural Practice‐based Research Network and the Kaiser Permanente Northwest Center for Health Research. Study activities built on prior research conducted by our team [9, 10, 16, 18, 24, 25, 26], best practices identified at a Centers for Disease Control Mailed FIT Summit [27], and participatory implementation science principles [28]. A study protocol provides more detail on study methods [18]. SMARTER CRC obtained approval from the Oregon Health & Science Institutional Review Board (protocol number: 20681). A ceding agreement was obtained from Kaiser Permanente Northwest Center for Health Research.

### 2.2 SMARTER CRC Intervention

SMARTER CRC used FIT, a self‐collection CRC screening modality that has been recommended by the US Preventive Services Task Force for average‐risk patients [1]. Patients who received an abnormal FIT result were referred for follow‐up colonoscopy for further evaluation and possible removal of polyps. Patient navigation provided in SMARTER CRC by primary care clinics and health plans was used to support completion of follow‐up colonoscopy.

**FIGURE 1:**
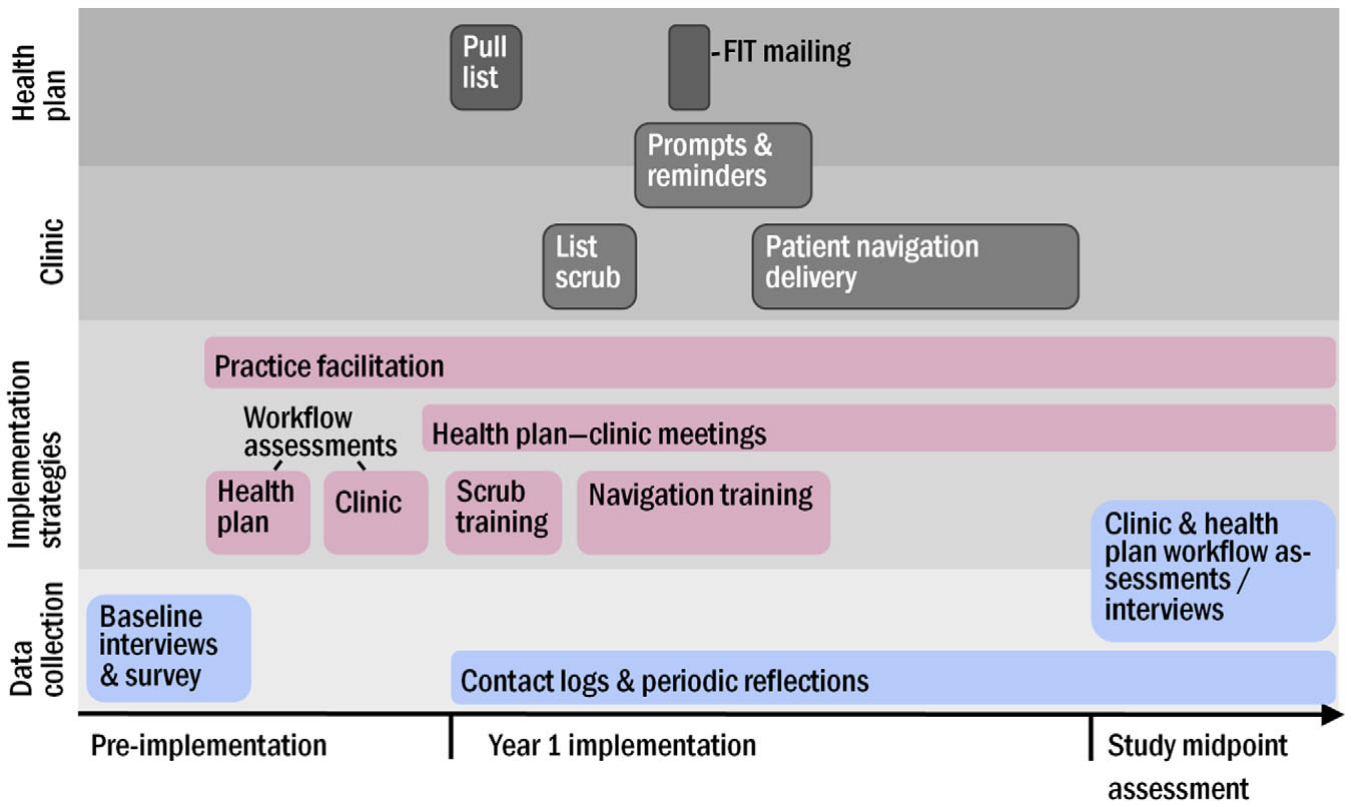
SMARTER CRC intervention, strategies, and data collection.

In the SMARTER CRC intervention, an initial workflow assessment enabled tailoring to health plan, clinic, and patient needs. Health plans selected mailing vendors, single versus clinic‐chosen FIT brand, and which resources it provided to clinics for patient outreach. Clinics further customized patient outreach and made plans for FIT selection, lab processing, staffing, and workflow for patient identification, tracking test completion, and navigating patients to colonoscopy. Before the mailing, health plans generated lists of patients due for CRC screening according to claims data. The clinics then “scrubbed” these lists to remove patients who did not have an address, had not been seen by the clinic, or did not meet eligibility criteria. Some health plans and clinics chose to send introductory letters or text messages to patients to orient them to the intervention. Health plans worked with vendors to mail FIT kits. Clinics and health plans were encouraged to conduct additional outreach to patients through reminder text messages or phone calls. 78.1% of patients received some kind of reminder after the FITs were mailed [9]. Clinic staff and one health plan staff member were trained to navigate patients who received abnormal FIT results. This navigation was intended to reduce barriers to follow‐up colonoscopy. Practice facilitation was used as a unifying meta‐strategy to support implementation [29, 30]. In the study protocol, planned practice facilitator responsibilities included maintaining ongoing contact with clinics, supporting training of clinic staff, conducting workflow assessments, and providing ad hoc implementation support [18]. We anticipated clinics to be the primary recipients of practice facilitation. Figure 1 outlines study components over time.

### 2.3 Data Collection

Data collection occurred from 2021 to 2022, concurrent with program preparation and the first year of study implementation. Study data include semi‐structured interviews conducted with health plan and clinic staff, clinic contact logs maintained by practice facilitators, and periodic reflections with practice facilitators conducted by qualitative analysts (E.K., E.M., and A.H.O.).

Semistructured interviews conducted at baseline were carried out with primary staff contacts of the 28 clinics and 3 health plans engaged in the research project. Interviewees were provided with an information sheet outlining details of the study and their rights as participants. Following the first year of implementation, practice facilitators conducted a brief qualitative interview with health plans and clinics as part of a workflow re‐assessment. When possible, the same staff were interviewed across implementation; however, staff turnover at clinics meant some turnover occurred between interviews. Interview guides were developed by qualitative analysts with input from topic area experts on mailed FIT and patient navigation, informed by established barriers and facilitators of implementation (staffing, funding, champions, etc.), and were aligned to the broader SMARTER CRC study goals. These guides were tailored for both clinic participants and health plans. Virtual interviews were conducted by trained qualitative analysts and lasted between 45 and 60 min. Audio was recorded via Zoom, transcribed through Rev.com, and validated by the analyst team.

Clinic contact logs were collected and managed using REDCap electronic data capture tools hosted at OHSU [31, 32]. These contact logs included field notes, next steps, and assessment of engagement. The logs were validated by a team analyst, and further information or clarifications were requested as they were posted. Separate meeting notes were taken summarizing interactions with health plans.

Periodic reflections were conducted throughout implementation (monthly when possible, but varied depending on study activity) with practice facilitators by a qualitative analyst [33]. In advance of these sessions, the qualitative analyst solicited discussion topics from study leadership and practice facilitators. These sessions served as an opportunity for facilitators to compare experiences and share perspectives not otherwise captured by the clinic contact logs. Audio was recorded via Zoom and transcribed through Rev.com and validated by the analyst team.

### 2.4 Data Analysis

We analyzed our data using an immersion crystallization approach [34]. Transcripts from all data sources were collected in ATLAS.ti for coding. A codebook was developed based on research questions and the interview guides. Example codes included: “Adaptations”, “CRC Screening_Outreach”, and “Rurality”. The analyst team, including three coders, came to consensus on the codebook and then co‐coded a subsection of the data to check for validity and coder agreement. Data were then split among the analyst group and coded.

Code reports were pulled and analyzed by the team. A subsection of each code report was analyzed by multiple analysts to ensure agreement among the team. Theme summaries were shared with the broader research team for feedback and member checking.

## 3 Results

### 3.1 Participant Profile

In total, 59 interviews were completed across all 3 health plans and 28 clinics. Staff from every participating clinic were interviewed at baseline and study midpoint. Interviewees were typically clinic staff (e.g., clinic manager, quality improvement staff) or providers. Details about SMARTER CRC clinic and health plan characteristics have been provided in prior publications [9, 26]. Practice facilitators recorded 214 contact log entries in REDCap and participated in 8 periodic reflection sessions. Over the first year of implementation, one practice facilitator left and was replaced. Four facilitators in total participated in data collection.

### 3.2 Overview of Themes

**FIGURE 2:**
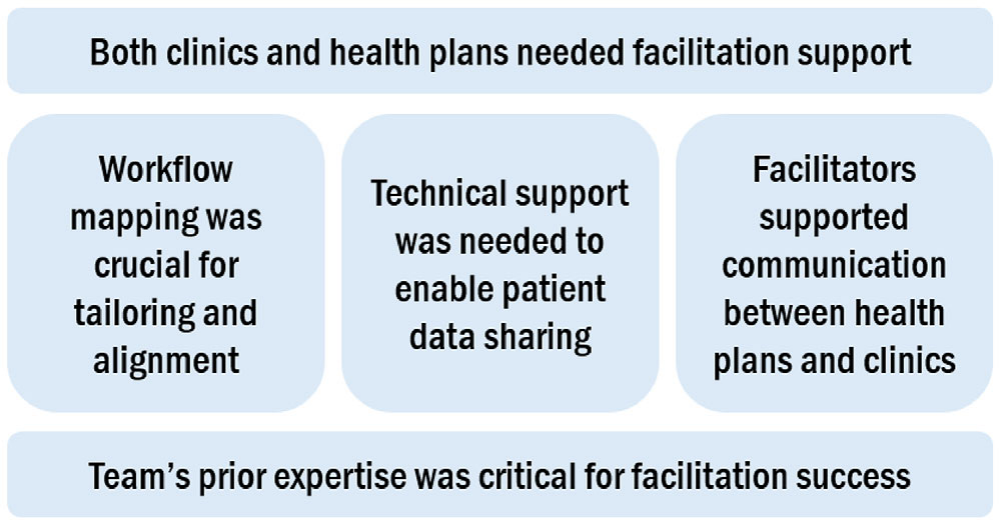
Overview of study themes.

Our analysis yielded five themes related to facilitation of health plan‐clinic partnerships in our study. Figure 2 provides an overview of these themes.

### 3.3 Both Clinics and Health Plans Needed Facilitation Support

The original SMARTER CRC study design included ongoing practice facilitation support for clinics, and a lighter degree of support for health plans (e.g., workflow mapping and subject‐matter experts for implementation questions). During implementation, it became apparent that a high degree of implementation support was needed for both clinics and health plans. Practice facilitators conducted workflow assessments with clinics; facilitated clinic training sessions on list scrubbing, outreach messaging, and follow‐up procedures; lent expertise regarding FIT selection; supported clinics with study data collection; and provided individualized support to clinics to tailor strategies to both clinic and patient needs. This expertise was identified by health plans as key to facilitating the intervention:

The [practice facilitators] definitely were helpful to have, to be able to coordinate with the clinics. You know, most of the clinics we had pretty good relationships with, so they know to come to us for other things, but it was nice to have another point of contact specific to this project who really was kind of that subject matter expert. ‐ Participant 2, Health plan 1

Practice facilitators helped keep clinics and health plans on task and engaged in the study during a time of significant external pressures due to the COVID‐19 pandemic. Facilitators described being strategic about spacing the number of requests made to clinics and health plans so as to be sensitive to external demands and avoid asking for too much at any given time. One facilitator describes this balance, saying, “You don't want to give them too much to overwhelm them, but then you don't want to say so little that they don't really know what they're agreeing to and what they're going to be asked to do.” In several cases, facilitators helped clinics sort out communication amongst their staff about the project. For example, one clinic manager was unaware that a provider signed their clinic up for the study.

Facilitators also supported clinics in tailoring outreach materials to patient needs. Several clinics requested help tailoring outreach materials and FIT instructions to specific patient populations (e.g., Spanish materials for Latinx patients, wordless or limited‐word instructions for patients with low English proficiency). Other clinics needed support finding ways to ensure the process for FIT distribution and return best met patient needs. For example, one clinic removed the optional Spanish language instructions and customized the letter to add Native American language phrases to tailor outreach to a primarily Tribal health population: “We should get feedback from nurses with strong community ties. [We] want to get our language teachers to include native language phrases on the card.”

### 3.4 Workflow Mapping Was Crucial for Tailoring Intervention and Alignment Between Clinic and Health Plan

Workflow mapping conducted before implementation served as an opportunity to tailor implementation and align activities between health plans and clinics. These sessions focused on decisions about mailing vendors, the patient data pull and clinic review, FIT selection, laboratory processing, patient outreach methods and timing, support available to clinics, and staff involved. After these decisions were made at the health plan level, the study team met with each clinic to review how their health plan was implementing the FIT mailing, discuss the workflow for collaborating with the health plan, review the lists, clinic‐level patient outreach, and patient navigation processes. Facilitators produced process flow diagrams, also called swimlane diagrams, summarizing clinic and health plan workflows to guide these discussions [35]. Decisions at the health plan level set the boundaries for decisions at the clinic level. For example, one clinic's plan (clinic 2) to scrub a large patient list in sections was altered when their partnered health plan required bulk mailing because of changes to postage costs at smaller bulk‐mail scales. This required the facilitators working with the clinic and health plan to identify a new patient list scrub timeline for the clinic that allowed for the largest bulk mail out possible.

For health plans and clinics that implemented the program in both years, a workflow reassessment meeting was conducted following the first year of implementation. At this meeting, study team members prompted health plans and clinics to discuss how the first year went and to identify any desired changes in workflow for the second year. For example, following challenging patient engagement, one clinic with primarily Tribal patients decided to change their outreach materials to be more culturally aligned. During this same workflow meeting, a decision was made to alter FIT return processing to account for cultural hesitancy by allowing for a dropbox collection method at the clinic.

### 3.5 Technical Support Was Needed to Enable Sharing of Patient Data

In SMARTER CRC, health plans compiled lists of patients due for CRC screening, which were given to clinics via the study team with the intention of streamlining identification of patients for the program. The question of which patients to include in CRC outreach was complicated, both in terms of which people were due for cancer screening and which people were being seen at which clinic. Although this was not a planned part of the intervention, the SMARTER CRC study team ended up providing technical support to health plans after noticing that health plans had questions about providing the requested information. This support included working with health plan data managers to identify appropriate criteria for screening eligibility, assessing data sets for quality, and cleaning data sets. Practice facilitators engaged clinics regarding their inclusion preferences. Specific technical recommendations were made to the clinics as well on accessing and handling data use agreements by the research team. With one health plan, they stored active and expired data in separate databases, which led to difficulty identifying the correct data until the research team worked directly with the health plan to identify the problem:

We didn't realize that [health plan 2] stores the “active most recent screening” and the “expired most recent screening” in two totally separate databases…and depending on the data request, they may or may not remember that. So, we would send a data request for that one variable, and we may or may not get things that recently expired and would have to go back to them and remind them. ‐ (Study team member 2)

### 3.6 Facilitators Supported Communication Between Health Plans and Clinics

Facilitators supported communication between clinics and health plans, which included connecting newly hired staff to health plan or clinic partners, outreaching to health plans on behalf of clinics, and ensuring alignment between health plans and clinics. Facilitators also supported clinic staff re‐orientation in the case of staff turnover to minimize disruption. Some facilitators described navigating internal clinic communications to ensure that the right people from a clinic were involved in decisions pertaining to study activities.

In one case, a practice facilitator reached out to the clinic's health plan to see if they could provide support making reminder phone calls. In another case, practice facilitators worked with health plans and clinics to identify and solve a lab processing issue related to the selection of a FIT kit:

Initially, [clinic] and [health plan] met to talk about which FIT kits they're going to be using. And they decided that we were going to use the OC‐Autos and would be provided by [health plan]. However, once [the research team and clinic] actually started talking about the actual implementation, we realized that the lab that [clinic] planned on having the FIT kits returned to didn't actually process the OC‐Autos. So we had to meet with them again and talk about what was feasible, and eventually we decided that it would be better for us to use the FIT kits that [clinic] usually uses. ‐ (Practice facilitator 1)

The primary touchpoint between the study team, clinics, and health plans was a monthly virtual meeting referred to as the health plan‐clinic meeting. This meeting provided an opportunity to orient health plans and clinics to different phases of the study and provide support for emergent issues. Meetings were facilitated by study leadership and practice facilitators. One clinic staff member communicated the value of these meetings from their perspective:

I didn't feel alone when we met as a larger group. I appreciated … that people were experiencing the same struggles we were and having some of the same feelings we had. So that was helpful for me and I appreciated that. ‐ (Participant 16)

### 3.7 Team's Prior Expertise Critical for Facilitation Success

The project manager and a co‐investigator, who both had prior experience with mailed FIT and patient navigation implementation, provided mentorship to practice facilitators and served as research team subject matter experts. They were the primary points of contact with health plans, trained practice facilitators, and helped troubleshoot facilitation challenges. This expertise was identified by facilitators as critical for intervention success among clinics. This expert research staff also acted as sources of information and advice for practice facilitators. This advice often related to handling complex communications with clinical sites, but also included specific technical knowledge of clinic agreements and options for alleviating clinic burden or confusion during the research project. In one example, the project manager offered advice to a facilitator on handling questions about second year program commitment from a clinic in order “to get a sense for how to best respond.”

## 4 Discussion

Partnerships between health plans and clinics are increasingly being used to support the implementation of evidence‐based clinical interventions like CRC screening. In this study, we used a health plan‐clinic partnership model to support implementation of mailed FIT and patient navigation for follow‐up colonoscopy in rural clinics serving Medicaid enrollees. While we anticipated that robust support from practice facilitators would be necessary for clinics, we planned on providing light facilitation for FIT mailing components to health plans. During implementation, we found that health plans also needed cross‐organizational facilitation and data support. Workflow mapping facilitated by the study team enabled alignment of strategies between health plans and clinics. Technical support was also needed to support sharing of patient data between health plans and clinics. Practice facilitators supported communication between health plans and clinics through monthly meetings and on an as‐needed basis. Finally, prior expertise of the study team leadership provided mentorship to novice facilitators and supported overall implementation success [36].

Our team chose a partnered model involving health plans for this study to overcome previously discovered barriers to implementing mailed FIT and patient navigation in rural settings [18]. Having health plans coordinate mailing logistics meant that postage and vendor relations could happen at an economy of scale not possible for individual clinics [9]. As found in this study, these efficiencies necessitated facilitation to support health plans and coordinate workflows and communication. This substantial role played by the study team facilitators raises questions about sustainability. Future research could examine how activities performed by facilitators in this study could be taken on or transferred to health plans or clinics, potentially using internal facilitators.

Many of the facilitation examples cited in our results required coordination and multiple conversations with clinics and health plans. For future preventive care interventions to be successful with a collaborative model, they need to build in sufficient time and an organizational lead to handle close collaboration with participating clinic partners.

Our research underscores the importance of subject‐matter expertise and mentorship to support and develop practice facilitators, aligning with prior research [37]. The practice facilitators demonstrated diverse and complex skills to support clinics, and were adept at guiding clinics through change, managing timelines, addressing implementation barriers, and holding clinics accountable [38]. Practice facilitators were able to build and sustain these skills through structured learning opportunities and coaching with study team leaders (i.e., project manager and co‐investigator), and through shadowing and consultation with more advanced peers [39]. Structured support allowed practice facilitators to subsequently support clinics and health plans through implementation of the complex multilevel and multicomponent SMARTER CRC intervention.

We conducted this study in Oregon, which has been described as an “ambitious” accountable care model in which in which Medicaid health plans assume financial risk for their patients, manage care, and are held accountable for the quality of care delivered through state‐defined quality metrics [40, 41]. In this system, Medicaid health plans (called coordinated care organizations) are incentivized to support patient outcomes by providing support to clinics [40]. Because CRC had been an incentive metric in the past, some precedent had been established for collaboration between health plans and clinics. While our findings are most directly generalizable to states with similar systems, two points are more broadly applicable. As has been shown in prior research [38, 42, 43], practice facilitation supported implementation by keeping clinic and health plan partners engaged, providing planning support through workflow mapping, providing technical assistance, and facilitating communication between partners. Our research also underscores the importance of assessing capabilities and support needs of all organizations when engaging multiple partners in implementation [10].

This study has several limitations. Because SMARTER CRC was conducted during the COVID‐19 pandemic, clinics, health plans, and study team members experienced significant external stressors [44]. These stressors included clinic and health plan staff turnover, clinic staffing shortages, and pandemic‐related care (e.g., vaccine campaigns) competing for limited staff time. While it is possible that facilitation needs for health plans or clinics might have been different without the pandemic during implementation, many of these stressors, such as sufficient staffing, are common challenges in primary care [8]. The qualitative data supporting this analysis were also only based on 1 year of implementation. Facilitation needs might change in subsequent years.

Future research should examine the long‐term impacts of health plan‐clinic interventions. For example, is the intervention sustained? It would be helpful to explore whether improved communication or relationships between health plans and clinics might result in increased collaboration on other preventive care efforts. Future studies could also examine how health plan‐clinic partnerships can be sustained in a way that is less reliant on practice facilitation support.

## 5 Conclusion

In the SMARTER CRC study, practice facilitation emerged as a critical meta‐strategy supporting health plan‐clinic partnerships to increase CRC screening in rural Medicaid populations. Facilitators provided essential guidance to both clinics and health plans by conducting workflow mapping, offering technical support for patient data sharing, facilitating cross‐organizational communication, and leveraging subject matter expertise to tailor the mailed FIT and patient navigation interventions. Subject‐matter expertise among study team leadership proved important for implementation success. These findings highlight the importance of assessing support needs across all partnering organizations when implementing multilevel preventive care interventions. Future research should investigate sustainability approaches focused on increasing clinic and health plan capacities and examine long‐term impacts of health plan‐clinic partnerships. This study demonstrates that even during a pandemic, strategic facilitation can effectively support complex implementation efforts that bridge organizational boundaries to improve preventive care access for underserved populations.

## Funding

This study was received funding from the National Cancer Institute: The Accelerating Colorectal Cancer Screening and Follow‐up through Implementation Science (ACCSIS) Program is a Beau Biden Cancer Moonsho^SM^ Initiative. Research was supported by the National Cancer Institute of the National Institutes of Health under Award #UH3CA244298, Screening More patients for CRC through Adapting and Refining Targeted Evidence‐based Interventions in Rural settings. The content is solely the responsibility of the authors and does not necessarily represent the official views of the NIH.

## Conflicts of Interest

The authors declare no conflicts of interest.
